# Outcomes of catheter ablation vs. medical treatment for atrial fibrillation and heart failure: a meta-analysis

**DOI:** 10.3389/fcvm.2023.1165011

**Published:** 2023-05-10

**Authors:** Wei-Chieh Lee, Hsiu-Yu Fang, Po-Jui Wu, Huang-Chung Chen, Yen-Nan Fang, Mien-Cheng Chen

**Affiliations:** ^1^ Institute of Clinical Medicine, College of Medicine, National Cheng Kung University, Tainan, Taiwan; ^2^ Division of Cardiology, Department of Internal Medicine, Chi Mei Medical Center, Tainan, Taiwan; ^3^ Division of Cardiology, Department of Internal Medicine, Kaohsiung Chang Gung Memorial Hospital, Chang Gung University College of Medicine, Kaohsiung, Taiwan; ^4^ Division of Cardiology, Department of Internal Medicine, National Cheng Kung University Hospital, College of Medicine, National Cheng Kung University, Tainan, Taiwan

**Keywords:** atrial fibrillation, heart failure, left ventricular ejection fraction, medical treatment, catheter ablation

## Abstract

**Background:**

The benefit of catheter ablation vs. medical treatment has been reported to be inconsistent in randomized controlled trials (RCTs) for patients with atrial fibrillation (AF) and heart failure (HF) due to different enrollment criteria. This meta-analysis aimed to decipher the differential outcomes stratified by different left ventricular ejection fractions (LVEFs) and AF types.

**Methods:**

We searched PubMed, Embase, ProQuest, ScienceDirect, Cochrane Library, ClinicalKey, Web of Science, and ClinicalTrials.gov databases for RCTs comparing medical treatment and catheter ablation in patients with AF and HF published before March 31, 2023. Nine studies were included.

**Results:**

When patients were stratified by LVEF, improved LVEF and 6-min walk distance, less AF recurrence, and lower all-cause mortality in favor of catheter ablation were observed in patients with LVEF ≤50% but not in patients with LVEF ≤35%, and short HF hospitalization was observed in patients with LVEF ≤50% and LVEF ≤35%. When patients were stratified by AF types, improved LVEF and 6-min walk distance, better HF questionnaire score, and short HF hospitalization in favor of catheter ablation were observed both in patients with nonparoxysmal AF and mixed AF (paroxysmal and persistent) and less AF recurrence and lower all-cause mortality in favor of catheter ablation were observed in only patients with mixed AF.

**Conclusions:**

This meta-analysis showed improved LVEF and 6-min walk distance, less AF recurrence, and lower all-cause mortality in favor of catheter ablation vs. medical treatment in AF patients with HF and LVEF of 36%–50%. Compared with medical treatment, catheter ablation improved LVEF and had better HF status in patients with nonparoxysmal AF and mixed AF; however, AF recurrence and all-cause mortality in favor of catheter ablation were observed in only HF patients with mixed AF.

## Introduction

The prevalence of atrial fibrillation (AF) and heart failure (HF) has increased globally (1). Advanced age and underlying structural heart disease are risk factors for AF and HF, which may develop sequentially or coincidentally (2). Patients with both conditions have worse outcomes and a higher risk of adverse clinical events (3, 4). Therefore, appropriate management of AF and HF is important to reduce morbidity and mortality in patients with AF and HF. One randomized controlled study showed that catheter ablation had a low reported rate of restoring sinus rhythm and did not improve N-terminal pro-B-type natriuretic peptide, 6-min walk distance, or quality of life in patients with persistent AF and HF when compared with rate control (5). However, several randomized controlled studies showed significant benefits from catheter ablation vs. rate control in terms of objective exercise performance, clinical symptoms, neurohormonal status, left ventricular ejection fraction (LVEF), unplanned hospitalization, and mortality in patients with persistent AF and HF (6–8). One randomized controlled trial (RCT) reported that catheter ablation was associated with a significantly lower rate of all-cause mortality or hospitalization for worsening HF than rhythm and rate control therapy in patients with paroxysmal and persistent AF and HF, especially in patients with LVEF ≥ 25% (9). One study reported that timely treatment of arrhythmia-mediated cardiomyopathy might minimize irreversible ventricular remodeling in patients with persistent AF and HF related to LV systolic dysfunction (LVEF ≤ 45%) (10). However, another RCT reported that catheter ablation did not improve LVEF compared with the best medical treatment in HF patients with persistent AF and LVEF ≤ 35% (11). In the subgroup analysis of the CABANA study, catheter ablation produced clinically important improvements in survival, freedom from AF recurrence, and quality of life compared with drug therapy in patients with paroxysmal or persistent AF and clinically stable heart failure with a mean LVEF of 55% (12). However, another open-label study showed no difference in all-cause mortality or HF events between catheter ablation and rate control in patients with high-burden paroxysmal AF or persistent AF and HF symptoms (13). Therefore, the benefit of catheter ablation vs. medical treatment has been reported to be inconsistent in patients with AF and HF regarding clinical symptoms and outcomes. The discrepancy in outcomes between catheter ablation and medical treatment in patients with AF and HF may be due to different inclusion criteria in terms of HF diagnostic criteria and LVEF and AF types. Therefore, this meta-analysis aimed to decipher the differential outcomes of catheter ablation vs. medical treatment in patients with AF and HF, stratified by different LVEFs, New York Heart Association (NYHA) class ≥ II, and different AF types.

## Methods

### Search strategies, trial selection, quality assessment, and data extraction

Two cardiologists (W-CL and H-YF) performed a systematic literature search of the PubMed, Embase, ProQuest, ScienceDirect, Cochrane Library, ClinicalKey, Web of Science, and ClinicalTrials.gov databases for articles published before March 31, 2023. The databases were searched for relevant studies without language restrictions using the key terms “atrial fibrillation,” “heart failure,” “catheter ablation,” and “medical treatment.” Disagreements were resolved by a third reviewer (P-JW). This study included different RCTs that compared the efficacies of catheter ablation and medical treatment in patients with AF and HF. The inclusion criteria were a human study with parallel design and comparison of the efficacy of catheter ablation and medical treatment in patients with AF and HF. The exclusion criteria were case reports or series, animal studies, review articles, conference abstracts, unpublished data, and observational studies. We did not set language limitations to increase the number of eligible articles. Supplementary Figure S1 illustrates the literature search and screening protocol.

### Outcomes

The outcomes of interest in this study were the change in LVEF, 6-min walk distance, HF questionnaire score, change in brain natriuretic peptide (BNP), AF recurrence, HF hospitalization, and all-cause mortality.

### Statistical analysis

The frequency of each evaluated outcome was extracted from each study, and the data were presented as cumulative rates. A random-effects model was employed to pool the individual odds ratio (OR), and all analyses were performed using Comprehensive Meta-Analysis software version 3 (Biostat, Englewood, NJ, United States). To assess the heterogeneity across trials, we used the chi-squared test (values of *p* ≤ 0.10 were considered significant) and *I*2 statistics to examine each outcome from low to high heterogeneity (25%–75%, respectively). Potential publication bias was assessed using Egger's test *via* funnel plots, and statistical significance was set at *p* ≤ 0.10. Statistical significance was set at *p* < 0.05 to compare the catheter ablation and medical treatment groups.

## Results

### Characteristics of included studies

The study selection process is illustrated in Supplementary Figure S1. Nine studies met our inclusion criteria. The study design, study period, participant characteristics, AF type, HF criteria, mean LVEF, and follow-up period are described in Table 1. A total of 2,074 participants (mean age, 65 ± 7.6 years; 70.9% men) were included. Most participants in these studies had nonparoxysmal AF (68%–100%). The enrollment criterion for HF trial patients in four studies was LVEF ≤ 35% (5, 6, 9, 11). In another three studies, different LVEF values were used to enroll HF patients, including ≤50% (7), ≤40% (8), and ≤45% (10). The remaining two studies did not declare the LVEF cutoff value for enrollment and used only a history of NYHA functional classification ≥II as the enrollment criteria (12, 13).

**Table 1:** Characteristics of the nine included studies.

First author (year)—study name	Patients number (male %)	Age (years)	Study period	Nonparoxysmal AF (%)	Enrollment criteria for HF	Mean LVEF of the two groups	Follow-up	Reference number
MacDonald MR (2011)	41 (78)	63 ± 7	January 2007–July 2009	100	NYHA class ≥ II and LVEF ≤ 35%	19.6 ± 5.5% vs. 16.1 ± 7.1%	6 months	5
Jones DG (2013)	52 (87)	63 ± 9	April 2009–June 2012	100	NYHA class ≥ II and LVEF ≤ 35%	25 ± 7% vs. 22 ± 8%	12 months	6
Hunter RJ (2014)—ARC AF	50 (96)	57 ± 11	N/A	92	NYHA class ≥ II and LVEF ≤ 50%	34 ± 12% vs. 32 ± 8%	12 months	7
Di Biase (2016)—CAMTAF	203 (74)	61 ± 11	N/A	100	NYHA class II, III, LVEF ≤ 40%, and an implanted defibrillator	30 ± 8% vs. 29 ± 5%	24 months	8
Marrouche NF (2018)—CASTLE-AF	363 (86)	64 ± 4	January 2008–January 2016	68	NYHA class ≥ II, LVEF ≤ 35%, and an implanted defibrillator	31.5 ± 2.5% vs. 32.5 ± 3.3%	37.8 months	9
Prabhu S (2018)—CAMERA MRI	36 (N/A)	61 ± 11	N/A	100	LVEF ≤ 45%	36 ± 8.2% vs. 33 ± 8.0%	6 months	10
Kuck KH (2021)—AMICA	140 (90)	65 ± 8	January 2008–June 2016	100	LVEF ≤ 35%	24.8 ± 8.8% vs. 27.8 ± 9.5%	12 months	11
Packer DL CABANA subgroup (2021)	778 (55)	67 ± 3	November 2009–April 2016	68	NYHA class ≥ II	56 ± 3% vs. 55 ± 3%	60 months	12
Parkash R (2022)—RAFTAF	411 (74)	67 ± 8	December 2011–January 2018	93	NYHA class II, III and elevated NT-proBNP	40.3 ± 14.6% vs. 41.0 ± 14.9%	24 months	13

HF, heart failure; AF, atrial fibrillation; LVEF, left ventricular ejection fraction; NYHA, New York Heart Association; N/A, not applicable; NT-proBNP; N-terminal pro-B-type natriuretic peptide.

### Patient demographics

Table 2 describes the details of patients’ demographics between the medical treatment and catheter ablation groups of the enrolled study patients. The mean age, sex, NYHA functional classification, prevalence of diabetes mellitus, hypertension, prior stroke, ischemic cardiomyopathy, implantable cardioverter defibrillator, cardiac resynchronization therapy placement, nonparoxysmal AF, AF duration, mean LVEF, and use of HF medications did not differ significantly between the medical treatment and catheter ablation groups.

**Table 2:** Patients’ demographics.

	Medical treatment	Catheter ablation	*P* value
Number	1,041	1,033	
Age (years)	65 ± 7.4 (1,041)	65 ± 7.8 (1,033)	1.000
Male sex (%)	71.5 (731/1,023)	70.3 (714/1,015)	0.551
Diabetes mellitus (%)	29.2 (289/991)	27.4 (269/981)	0.375
Hypertension (%)	73.4 (745/1,015)	71.5 (720/1,007)	0.339
Nonparoxysmal AF (%)	79.1 (808/1,022)	81.8 (827/1,011)	0.125
ICM (%)	41.0 (214/522)	37.9 (203/535)	0.303
Coronary artery disease (%)	31.6 (293/927)	30.9 (285/921)	0.746
Prior stroke (%)	10.3 (84/818)	10.5 (85/811)	0.895
NYHA class ≥ III (%)	39.1 (204/522)	42.8 (229/535)	0.222
ICD and CRT (%)	57.2 (274/479)	57.1 (278/487)	0.975
LA dimension (mm)	48.2 ± 6.3 (548)	47.6 ± 6.4 (563)	0.116
LVEF (%)	41.7 ± 14.9 (1,041)	41.2 ± 14.1 (1,033)	0.433
CHA2DS2-VASc score	3.1 ± 1.2 (615)	3.1 ± 1.2 (610)	1.000
AF duration (months)	16.6 ± 21.3 (785)	15.7 ± 16.1 (786)	0.345
ACEI/ARB (%)	88.3 (545/617)	86.2 (542/629)	0.267
*β* -blocker (%)	84.4 (521/617)	82.7 (520/629)	0.419
MRA (%)	59.0 (364/617)	60.9 (383/629)	0.494

AF, atrial fibrillation; ICM, ischemic cardiomyopathy; NYHA, New York Heart Association; ICD, implantable cardioverter defibrillator; CRT, cardiac resynchronization therapy; LA, left atrium; LVEF, left ventricular ejection fraction; ACEI, angiotensin-converting enzyme inhibitor; ARB, angiotensin II receptor blocker; MRA, mineralocorticoid receptor antagonist.

Data are expressed as mean ± SD or number (percentage).

### Pooled results of changes in LVEF, 6-min walk distance, HF questionnaire score, BNP level, AF recurrence, HF hospitalization, and all-cause mortality

**Figure 1:**
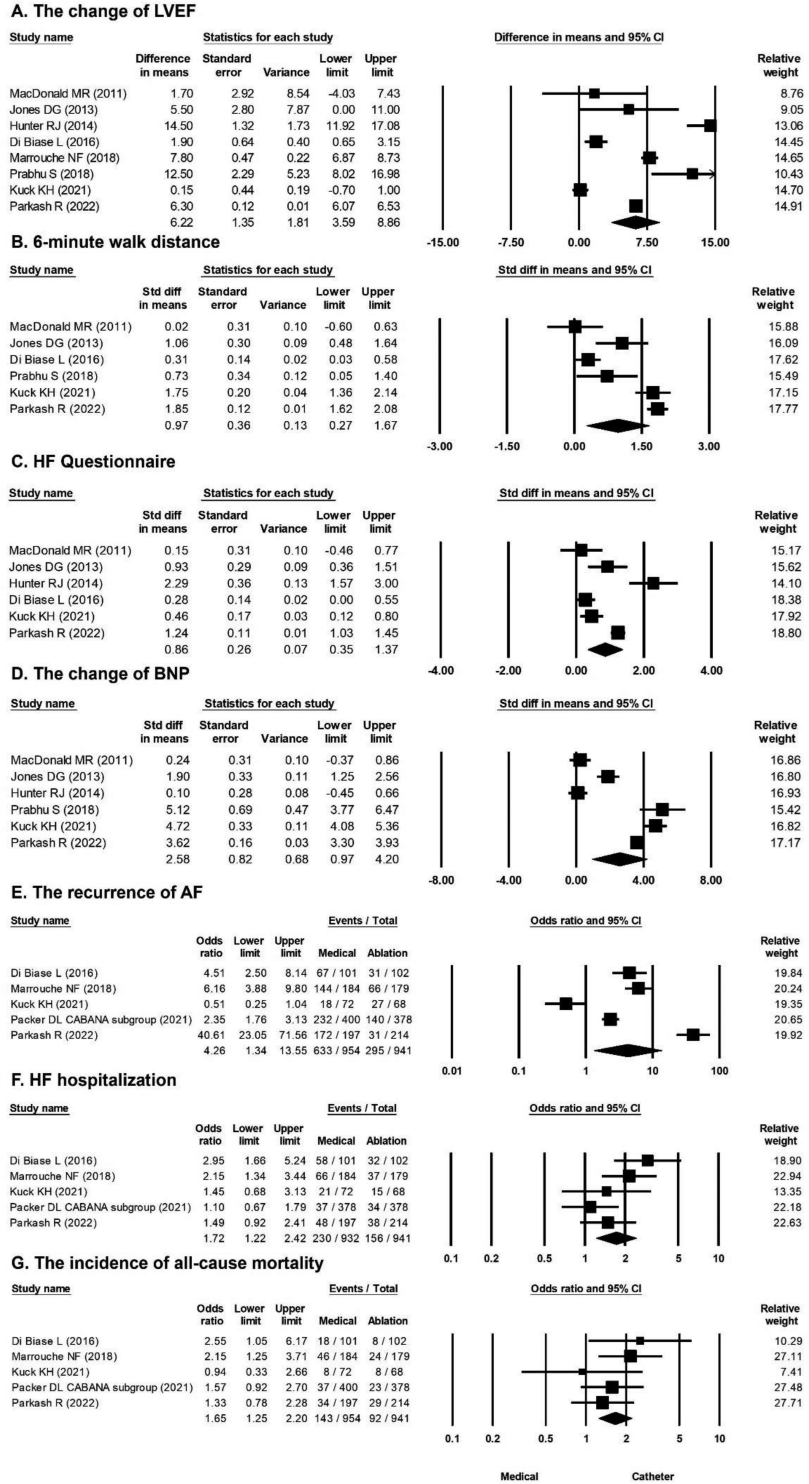
Forest plots comparing the changes in LVEF, 6-min walk distance, HF questionnaire score, BNP level, odds ratio for AF recurrence, odds ratio for HF hospitalization, and odds ratio for all-cause mortality of medical treatment versus catheter ablation. (**A**) Change of LVEF in eight studies. (**B**) 6-min walk distance in six studies. (**C**) HF questionnaire in six studies. (**D**) Change of BNP level in six studies. (**E**) AF recurrence rate in five studies. (**F**) HF hospitalization rate in five studies. (**G**) All-cause mortality rate in five studies. AF, atrial fibrillation; BNP, B-type natriuretic peptide; HF, heart failure; LVEF, left ventricular ejection fraction.

Pooled results from the random-effects model showed that catheter ablation for AF, compared with medical treatment, was associated with an increased LVEF from baseline [mean difference 6.22%; 95% confidence interval (CI), 3.59%–8.86%] with high heterogeneity (Cochran's *Q*, 294.657; *df*, 7; *I*^2^, 97.624%; *p *< 0.001) (Figure 1A). Egger's test revealed nonsignificant publication bias in the change in LVEF (*t*, 0.309; *df*, 6; *p *= 0.767). The funnel plot of the change in LVEF is shown in Supplementary Figure S2. Pooled results from the random-effects model showed that catheter ablation vs. medical treatment was associated with an increased 6-min walk distance from baseline (mean difference, 0.97 m; 95% CI, 0.27–1.67 m), with high heterogeneity (Cochran's *Q*, 94.559; *df*, 5; *I*^2^, 94.712%; *p *< 0.001) (Figure 1B). Egger's test revealed a nonsignificant publication bias regarding the change in the 6-min walk distance (*t*, 0.782; *df*, 4; *p *= 0.478). The funnel plot for the change in the 6-min walk distance is shown in Supplementary Figure S3. Pooled results from the random-effects model showed that catheter ablation vs. medical therapy was associated with an improved HF questionnaire score from baseline (mean difference, 0.86; 95% CI, 0.35–1.37) with high heterogeneity (Cochran's *Q*, 55.150; *df*, 5; *I*^2^, 90.934%; *p *< 0.001) (Figure 1C). Egger's test showed a nonsignificant publication bias in the change in the HF questionnaire score (*t*, 0.028; *df*, 4; *p *= 0.979). The funnel plot for the change in HF questionnaire score is shown in Supplementary Figure S4. Pooled results from the random-effects model showed that catheter ablation vs. medical treatment was associated with significant change in the BNP level from baseline (mean difference, 2.58 pg/ml, 95% CI, 0.97–4.20 pg/ml) with high heterogeneity (Cochran's *Q*, 233.478; *df*, 5; *I*^2^, 97.858%; *p *< 0.001) (Figure 1D). Egger's test revealed a nonsignificant publication bias for the change in the BNP level (*t,* 0.392; *df*, 4; *p *= 0.715). The funnel plot for the change in the BNP level is shown in Supplementary Figure S5. The overall OR of the recurrence of AF of the catheter ablation group vs. medical treatment was 4.26 (95% CI, 1.34–13.55) in favor of catheter ablation (Figure 1E) with high heterogeneity (Cochran's *Q*, 112.389; *df*, 4; *I*^2^, 96.441%; *p *< 0.001). Egger's test revealed nonsignificant publication bias regarding the overall OR of AF recurrence (*t,* 0.382; *df*, 3; *p *= 0.728). A funnel plot for the log OR of AF recurrence is shown in Supplementary Figure S6. The overall OR of the HF hospitalization of catheter ablation vs. medical treatment was 1.72 (95% CI, 1.22–2.42) in favor of catheter ablation (Figure 1F) with moderate heterogeneity (Cochran's *Q*, 7.991; *df*, 4; *I*^2^, 49.946%; *p *= 0.092). Egger's test revealed a nonsignificant publication bias regarding the overall OR of hospitalization for HF (*t,* 0.180; *df*, 3; *p *= 0.869). A funnel plot for the log OR of HF hospitalization is shown in Supplementary Figure S7. The overall OR of the incidence of all-cause mortality of catheter ablation vs. medical treatment was 1.65 (95% CI, 1.25–2.20) in favor of catheter ablation (Figure 1G) with low heterogeneity (Cochran's *Q*, 3.622; *df*, 4; *I*^2^, 0%; *p *= 0.460). Egger's test revealed a nonsignificant publication bias regarding the overall OR of all-cause mortality (*t,* 0.215; *df*, 3; *p *= 0.844). The funnel plot for the log OR of all-cause mortality is shown in Supplementary Figure S8.

### Pooled results of change in LVEF, 6-min walk distance, HF questionnaire score, BNP level, AF recurrence, HF hospitalization, and all-cause mortality stratified by different LVEFs

**Figure 2:**
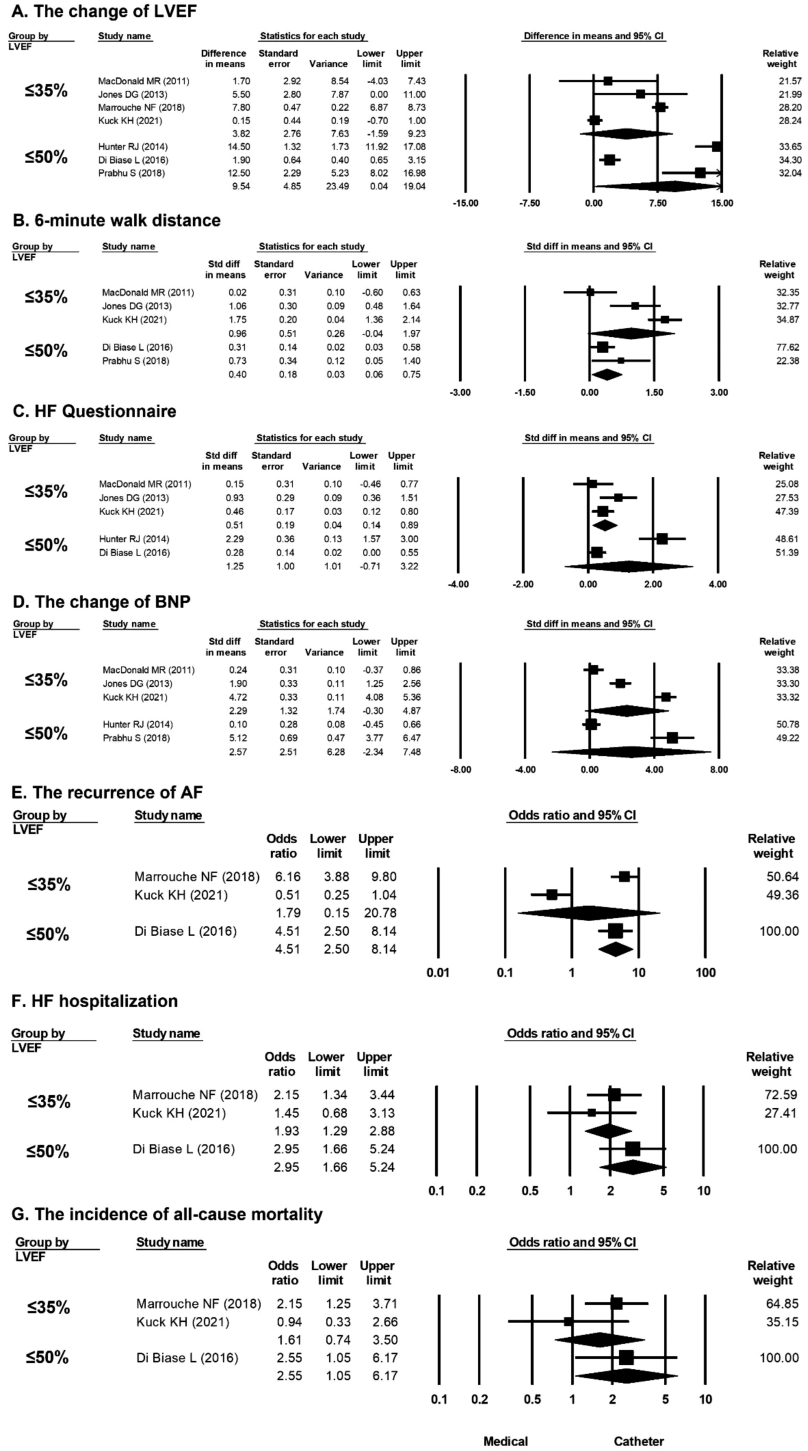
Forest plots comparing the changes in LVEF, 6-min walk distance, HF questionnaire score, BNP level, odds ratio for AF recurrence, odds ratio for HF hospitalization, and odds ratio for all-cause mortality of medical treatment versus catheter ablation in patients stratified by LVEF (LVEF ≤ 35% and LVEF ≤ 50%). (**A**) Change in LVEF in seven studies (≤35% in four, ≤50% in three). (**B**) 6-min walk distance in five studies (LVEF ≤ 35% in three, LVEF ≤ 50% in two). (**C**) HF questionnaire score in five studies (LVEF ≤ 35% in three, LVEF ≤ 50% in two). (**D**) BNP level in five studies (LVEF ≤ 35% in three, LVEF ≤ 50% in two). (**E**) AF recurrence in three studies (LVEF ≤ 35% in two, LVEF ≤ 50% in one). (**F**) HF hospitalization in three studies (LVEF ≤ 35% in two, LVEF ≤ 50% in one). (**G**) All-cause mortality rate in three studies (LVEF ≤ 35% in two, LVEF ≤ 50% in one). AF, atrial fibrillation; BNP, B-type natriuretic peptide; HF, heart failure; LVEF, left ventricular ejection fraction.

A greater improvement in LVEF in favor of catheter ablation vs. medical treatment was observed in the population with LVEF ≤50% (mean difference, 9.54%; 95% CI, 0.04%–19.04%) but not in the population with LVEF ≤35% (Figure 2A). A longer 6-min walk distance in favor of catheter ablation vs. medical treatment was observed in the population with LVEF ≤50% (mean difference, 0.40 m; 95% CI, 0.03–0.75 m), but not in the population with LVEF ≤35% (Figure 2B). Interestingly, a greater improvement in HF questionnaire scores in favor of catheter ablation vs. medical treatment was observed in the population with LVEF ≤35% (mean difference, 0.51; 95% CI, 0.14–0.89) but not in the population with LVEF ≤50% (Figure 2C). There was no significant difference in the change in the BNP level between catheter ablation and medical treatment in the population with LVEF ≤35% (mean difference, 2.29 pg/ml; 95% CI, −0.30 to 4.87 pg/ml) and in the population with LVEF ≤50% (mean difference, 2.57 pg/ml; 95% CI, −2.34 to 7.48 pg/ml) (Figure 2D). The risk of recurrence of AF was significantly lower by catheter ablation compared with medical treatment in the population with LVEF ≤50% (OR, 4.51; 95% CI, 2.50–8.14) but not in the population with LVEF ≤35% (Figure 2E). The overall OR values of HF hospitalization were 1.93 (95% CI, 1.29–2.88), in favor of catheter ablation vs. medical treatment in the population with LVEF ≤35%, and 2.95 (95% CI, 1.66–5.24), also in favor of catheter ablation vs. medical treatment in the population with LVEF ≤50% (Figure 2F). The incidence of all-cause mortality was significantly lower by catheter ablation compared with medical treatment in the population with LVEF ≤50% (OR, 2.55; 95% CI, 1.05–6.17) but not in the population with LVEF ≤35% (Figure 2G).

### Pooled results of change in LVEF, 6-min walk distance, HF questionnaire score, BNP level, AF recurrence, HF hospitalization, and all-cause mortality stratified only by NYHA ≥II

**Figure 3:**
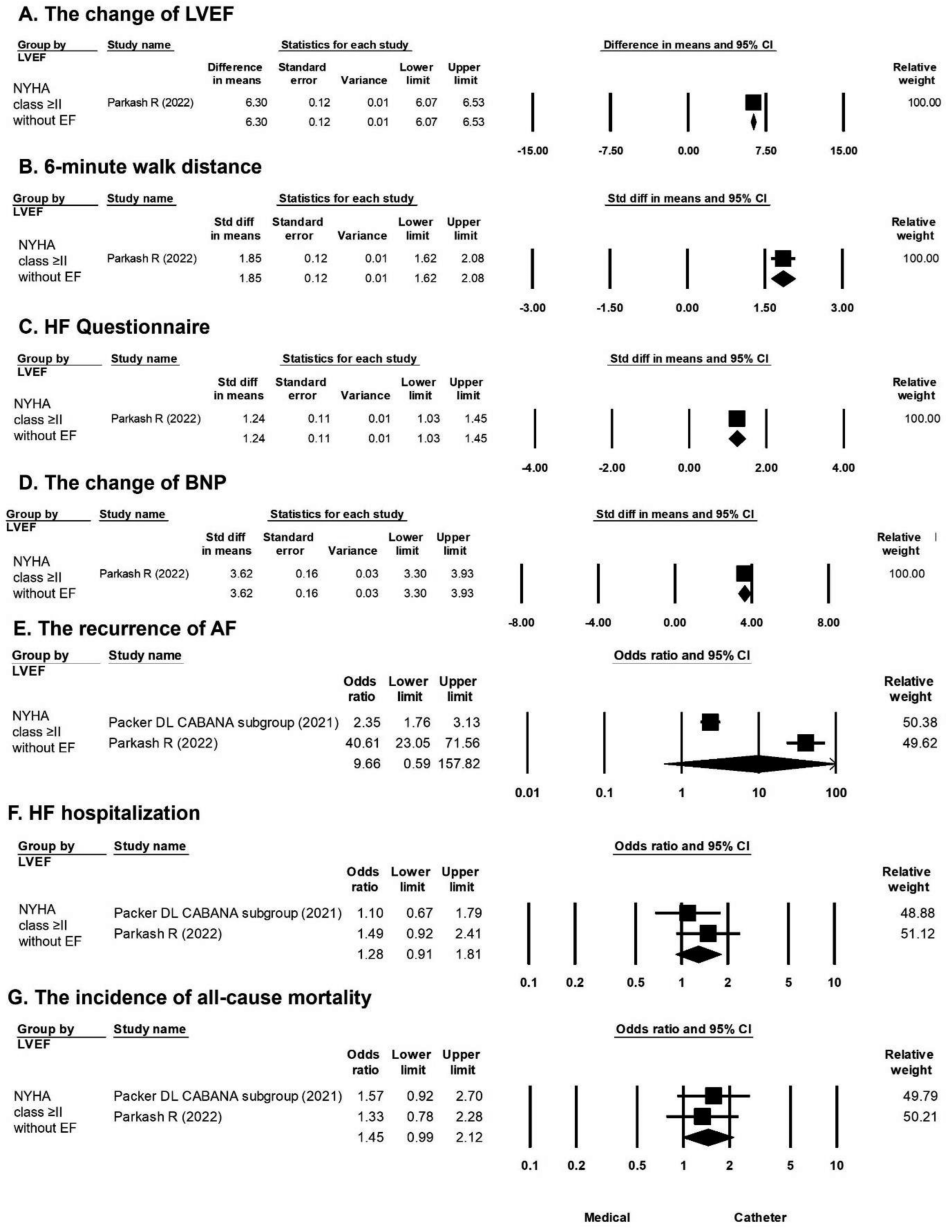
Forest plots comparing the changes in LVEF, 6-min walk distance, HF questionnaire score, BNP level, odds ratio for AF recurrence, odds ratio for HF hospitalization, and odds ratio for all-cause mortality of medical treatment versus catheter ablation in patients stratified by NYHA ≥ II without LVEF. (**A**) Change in LVEF in one study. (**B**) 6-min walk distance in one study. (**C**) HF questionnaire score in one study. (**D**) BNP level in one study. (**E**) AF recurrence in two studies. (**F**) HF hospitalization in two studies. (**G**) All-cause mortality rate in two studies. AF, atrial fibrillation; BNP, B-type natriuretic peptide; HF, heart failure; LVEF, left ventricular ejection fraction.

A greater improvement in LVEF in favor of catheter ablation vs. medical treatment was observed in the population with HF history (mean difference, 6.30%; 95% CI, 6.07%–6.53%) (Figure 3A). A longer 6-min walk distance in favor of catheter ablation vs. medical treatment was observed in the population with HF history (mean difference, 1.85 m; 95% CI, 1.62–2.08 m) (Figure 3B). A greater improvement in HF questionnaire scores in favor of catheter ablation vs. medical treatment was observed in the population with HF history (mean difference, 1.24; 95% CI, 1.03–1.45) (Figure 3C). A significant difference in the change in the BNP level in favor of catheter ablation vs. medical treatment was observed in the population with HF history (mean difference, 3.62 pg/ml; 95% CI, 3.30–3.93 pg/ml) (Figure 3D). There was no significant difference in the recurrence of AF, HF hospitalization, and all-cause mortality between catheter ablation and medical treatment in the population with HF history of NYHA ≥II (Figures 3E–G).

### Pooled results of change in LVEF, 6-min walk distance, HF questionnaire score, BNP level, AF recurrence, HF hospitalization, and all-cause mortality stratified by AF types

**Figure 4:**
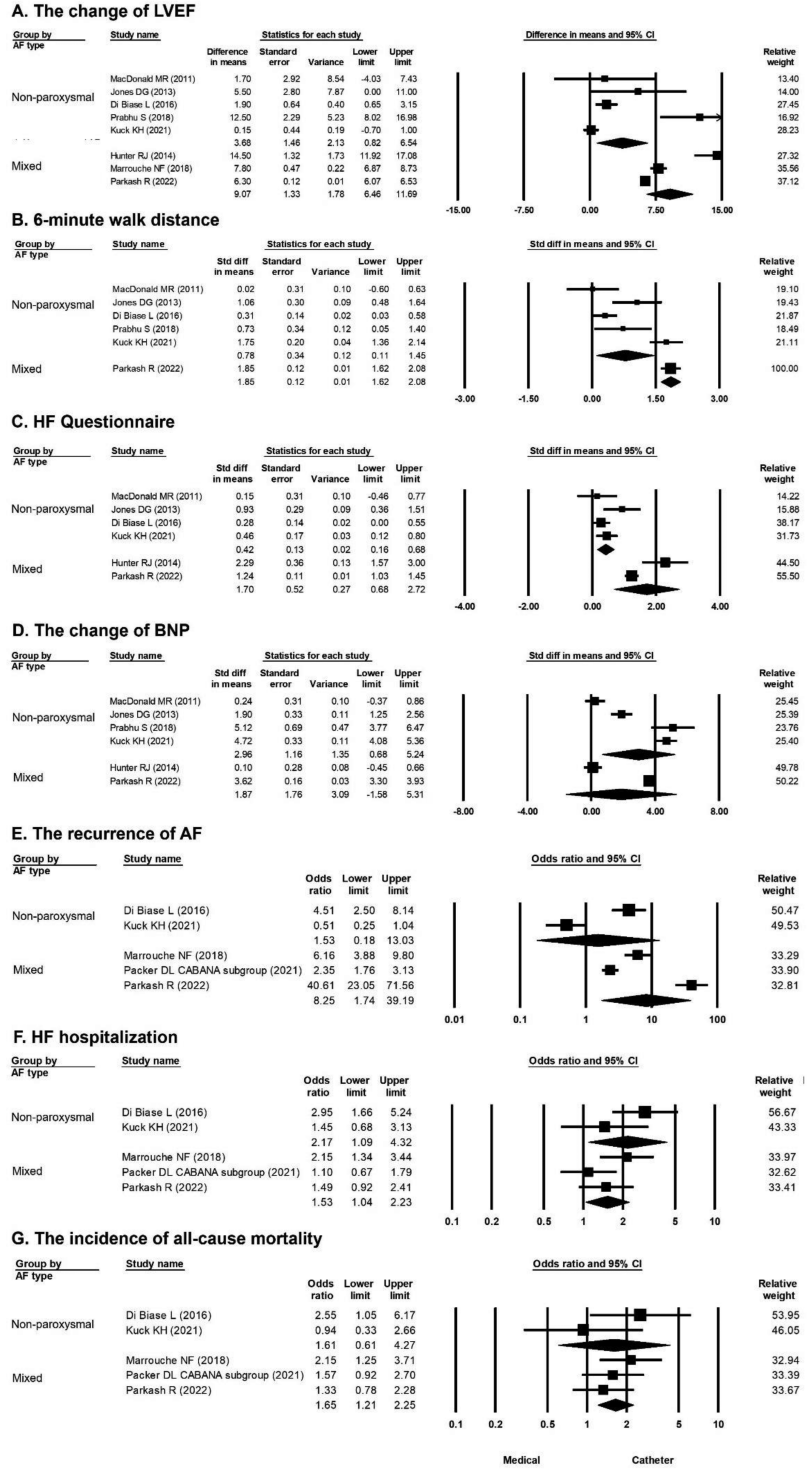
Forest plots comparing the changes of LVEF, 6-min walk distance, HF questionnaire score, BNP level, odds ratio for AF recurrence, odds ratio for HF hospitalization, and odds ratio for all-cause mortality of medical treatment versus catheter ablation in patients stratified by different AF types (nonparoxysmal and mixed AF). (**A**) Change in LVEF in eight studies (nonparoxysmal AF in five, mixed AF in three). (**B**) 6-min walk distance in six studies (nonparoxysmal AF in five, mixed AF in one). (**C**) HF questionnaire score in six studies (nonparoxysmal AF in four, mixed AF in two). (**D**) BNP level in six studies (nonparoxysmal AF in four, mixed AF in two). (**E**) AF recurrence rate in five studies (nonparoxysmal AF in two, mixed AF in three). (**F**) HF hospitalization rate in five studies (nonparoxysmal AF in two, mixed AF in three). (**G**) All-cause mortality rate in five studies (nonparoxysmal AF in two, mixed AF in three). AF, atrial fibrillation; BNP, B-type natriuretic peptide; HF, heart failure; LVEF, left ventricular ejection fraction.

Mixed AF was defined as the study population with paroxysmal and persistent AF (14). A greater improvement in LVEF in favor of catheter ablation vs. medical treatment was observed in the population with nonparoxysmal AF (mean difference, 3.68%; 95% CI, 0.82%–6.54%) and mixed AF (mean difference, 9.07%; 95% CI, 6.46%–11.69%) (Figure 4A). A longer 6-min walk distance in favor of catheter ablation vs. medical treatment was observed in the population with nonparoxysmal AF (mean difference, 0.78 m; 95% CI, 0.11–1.45 m) and mixed AF (mean difference, 1.85 m; 95% CI, 1.62–2.08 m) (Figure 4B). A greater improvement in HF questionnaire scores in favor of catheter ablation vs. medical treatment was observed in the population with nonparoxysmal AF (mean difference, 0.42; 95% CI, 0.16–0.68) and mixed AF (mean difference, 1.70; 95% CI, 0.68–2.72) (Figure 4C). A significant difference in the change in the BNP level in favor of catheter ablation vs. medical treatment was observed in the population with nonparoxysmal AF (mean difference, 2.96 pg/ml; 95% CI, 0.68–5.24 pg/ml) but not in the population with mixed AF (Figure 4D). The risk of recurrence of AF was significantly lower by catheter ablation compared with medical treatment in the population with mixed AF (OR, 8.25; 95% CI, 1.74–39.19) but not in the population with nonparoxysmal AF (Figure 4E). The overall OR values of HF hospitalization in favor of catheter ablation vs. medical treatment were 2.17 (95% CI, 1.09–4.32) in the population with nonparoxysmal AF and 1.53 (95% CI, 1.04–2.23) in the population with mixed AF (Figure 4F). The incidence of all-cause mortality was significantly lower by catheter ablation compared with medical treatment in the population with mixed AF (OR, 1.65; 95% CI, 1.21–2.25) but not in the population with nonparoxysmal AF (Figure 4G).

## Discussion

In the whole study population of this meta-analysis, improved LVEF, improved 6-min walk distance, better HF questionnaire score, significantly decreased BNP level, less AF recurrence, less HF hospitalization, and lower all-cause mortality were observed after catheter ablation vs. medical treatment. When the study population was stratified by LVEF, improved LVEF, improved 6-min walk distance, less AF recurrence, and lower all-cause mortality in favor of catheter ablation vs. medical treatment were observed in the population with LVEF ≤50% but not in the population with LVEF ≤35%; however, less HF hospitalization was observed both in the population with LVEF ≤50% and LVEF ≤35%. When the study population was stratified by HF NYHA ≥II, improved LVEF, improved 6-min walk distance, and better HF questionnaire score in favor of catheter ablation vs. medical treatment were observed in the population with HF NYHA ≥II. When the study population was stratified by AF types, improved LVEF, improved 6-min walk distance, better HF questionnaire score, and less HF hospitalization in favor of catheter ablation vs. medical treatment were observed both in the population with nonparoxysmal AF and mixed AF; however, less AF recurrence and lower all-cause mortality in favor of catheter ablation vs. medical treatment were observed only in the population with mixed AF.

### Population stratified by different LVEF criteria

The criteria for HF in the enrolled studies differed in LVEF cutoff values, ranging from LVEF ≤35% (5, 6, 9, 11) to ≤40% (8), ≤45% (10), and ≤50% (7) or differed in only enrolling patients with a history of HF with NYHA functional classification ≥II without mention LVEF (12, 13). According to our meta-analysis, improved LVEF, improved 6-min walk distance, less AF recurrence, and lower all-cause mortality in favor of catheter ablation vs. medical treatment were observed in the population with LVEF of 36%–50% and less HF hospitalization was observed both in the population with LVEF ≤ 50%, and LVEF ≤35%.

### Population stratified by different AF types

In patients with HF and reduced LVEF, a high prevalence of persistent AF exists and is closely related to underlying heart disease severity and HF functional classes (15). In the enrolled studies of this meta-analysis, the prevalence of nonparoxysmal AF was 68%–100%. Previous meta-analyses comparing catheter ablation vs. medical treatment in terms of clinical outcomes in patients with AF and HF did not specifically stratify the study subjects by different AF types (16, 17). However, the long-term efficacy of catheter ablation vs. medical treatment for different AF types on clinical outcomes may differ and may require more than one catheter ablation procedure for different AF types (18). In this meta-analysis, improved LVEF, improved 6-min walk distance, better HF questionnaire score, and less HF hospitalization in favor of catheter ablation vs. medical treatment were observed both in the population with nonparoxysmal AF and mixed AF; however, AF recurrence and all-cause mortality in favor of catheter ablation vs. medical treatment were only observed in the population with mixed AF but not in the population with nonparoxysmal AF. Nonparoxysmal AF may contribute to more atrial and ventricular structural remodeling and atrial fibrosis, reducing the benefit of catheter ablation for AF, especially in HF patients. Therefore, catheter ablation could achieve more clinical benefits in patients with mixed AF than in patients with nonparoxysmal AF.

### Limitations

This study had several limitations. First, the enrollment criteria for HF differed among the nine enrolled studies, and high heterogeneity was found in the analyses of the whole population. Therefore, we performed subgroup analyses, and patients were stratified by different LVEFs, HF history of NYHA ≥ II, and AF types. Second, the use of HF biomarkers differed among six studies, three (6, 7, 10) used serum BNP and the other three (5, 11, 13) used N-terminal proBNP. Third, although nine studies were included, over one-third of the 2,074 participants enrolled in this meta-analysis were derived from the HF subgroup of the CABANA study, which contributes a large number of patients with LVEF >50% (12). Fourth, the baseline characteristics of all participants in the enrolled studies were not completely available. Fifth, the enrolled studies had different follow-up periods, while HF hospitalization and all-cause mortality might need longer follow-up periods to show a significant difference between catheter ablation and medical treatment.

## Conclusion

This meta-analysis showed improved LVEF, improved 6-min walk distance, less AF recurrence, and lower all-cause mortality in favor of catheter ablation vs. medical treatment in AF patients with HF and LVEF of 36%–50%, and less HF hospitalization was observed both in AF patients with HF and LVEF ≤50%, and LVEF ≤35%. Compared with medical treatment, catheter ablation improved LVEF, improved 6-min walk distance, and had better HF questionnaire score and less HF hospitalization in patients with nonparoxysmal AF and mixed AF; however, AF recurrence and all-cause mortality in favor of catheter ablation were observed only in HF patients with mixed AF.

## Data Availability

The original contributions presented in the study are included in the article/**Supplementary Material**, further inquiries can be directed to the corresponding author/s.

## Supplementary material

SUPPLEMENTARY FIGURE 1

Flowchart of the study selection strategy and inclusion and exclusion criteria for this meta-analysis. AF, atrial fibrillation; HF, heart failure

SUPPLEMENTARY FIGURE 2

Funnel plot showing non-significant publication bias using Egger's test (*t*, 0.309; *df*, 6; *p* = 0.767).

SUPPLEMENTARY FIGURE 3

Funnel plot showing non-significant publication bias using Egger's test (n *t*, 0.782; *df*, 4; *p* = 0.478).

SUPPLEMENTARY FIGURE 4

Funnel plot showing non-significant publication bias using Egger's test (*t*, 0.028; *df*, 4; *p* = 0.979).

SUPPLEMENTARY FIGURE 5

Funnel plot showing non-significant publication bias using Egger's test (*t*, 0.392; *df*, 4; *p* = 0.715).

SUPPLEMENTARY FIGURE 6

Funnel plot showing non-significant publication bias using Egger's test (*t*, 0.382; *df*, 3; *p* = 0.728).

SUPPLEMENTARY FIGURE 7

Funnel plot showing non-significant publication bias using Egger's test (*t*, 0.180; *df*, 3; *p* = 0.869).

SUPPLEMENTARY FIGURE 8

Funnel plot showing non-significant publication bias using Egger's test (*t*, 0.215; *df*, 3; *p* = 0.844).

## References

[1] Anter E Jessup M Callans DJ. Atrial fibrillation and heart failure: treatment considerations for a dual epidemic. Circulation. (2009) 119(18):2516–25. 10.1161/CIRCULATIONAHA.108.821306 19433768

[2] Heck PM Lee JM Kistler PM. Atrial fibrillation in heart failure in the older population. Heart Fail Clin. (2013) 9(4):451–9, viii–ix. 10.1016/j.hfc.2013.07.007 24054478

[3] Batul SA Gopinathannair R. Atrial fibrillation in heart failure: a therapeutic challenge of our times. Korean Circ J. (2017) 47(5):644–62. 10.4070/kcj.2017.0040 28955382 PMC5614940

[4] Gopinathannair R Chen LY Chung MK Cornwell WK Furie KL Lakkireddy DR Managing atrial fibrillation in patients with heart failure and reduced ejection fraction: a scientific statement from the American heart association. Circ Arrhythm Electrophysiol. (2021) 14(6):HAE0000000000000078. 10.1161/HAE.0000000000000078 34129347

[5] MacDonald MR Connelly DT Hawkins NM Steedman T Payne J Shaw M Radiofrequency ablation for persistent atrial fibrillation in patients with advanced heart failure and severe left ventricular systolic dysfunction: a randomised controlled trial. Heart. (2011) 97(9):740–7. 10.1136/hrt.2010.207340 21051458

[6] Jones DG Haldar SK Hussain W Sharma R Francis DP Rahman-Haley SL A randomized trial to assess catheter ablation versus rate control in the management of persistent atrial fibrillation in heart failure. J Am Coll Cardiol. (2013) 61(18):1894–903. 10.1016/j.jacc.2013.01.069 23500267

[7] Hunter RJ Berriman TJ Diab I Kamdar R Richmond L Baker V A randomized controlled trial of catheter ablation versus medical treatment of atrial fibrillation in heart failure (the CAMTAF trial). Circ Arrhythm Electrophysiol. (2014) 7(1):31–8. 10.1161/CIRCEP.113.000806 24382410

[8] Di Biase L Mohanty P Mohanty S Santangeli P Trivedi C Lakkireddy D Ablation versus amiodarone for treatment of persistent atrial fibrillation in patients with congestive heart failure and an implanted device: results from the AATAC multicenter randomized trial. Circulation. (2016) 133(17):1637–44. 10.1161/CIRCULATIONAHA.115.019406 27029350

[9] Marrouche NF Brachmann J Andresen D Siebels J Boersma L Jordaens L Catheter ablation for atrial fibrillation with heart failure. N Engl J Med. (2018) 378(5):417–27. 10.1056/NEJMoa1707855 29385358

[10] Prabhu S Costello BT Taylor AJ Gutman SJ Voskoboinik A McLellan AJA Regression of diffuse ventricular fibrosis following restoration of sinus rhythm with catheter ablation in patients with atrial fibrillation and systolic dysfunction: a substudy of the CAMERA MRI trial. JACC Clin Electrophysiol. (2018) 4(8):999–1007. 10.1016/j.jacep.2018.04.013 30139501

[11] Kuck KH Merkely B Zahn R Arentz T Seidl K Schlüter M Catheter ablation versus best medical therapy in patients with persistent atrial fibrillation and congestive heart failure: the randomized AMICA trial. Circ Arrhythm Electrophysiol. (2019) 12(12):e007731. 10.1161/CIRCEP.119.007731 31760819

[12] Packer DL Piccini JP Monahan KH Al-Khalidi HR Silverstein AP Noseworthy PA Ablation versus drug therapy for atrial fibrillation in heart failure: results from the CABANA trial. Circulation. (2021) 143(14):1377–90. 10.1161/CIRCULATIONAHA.120.050991 33554614 PMC8030730

[13] Parkash R Wells GA Rouleau J Talajic M Essebag V Skanes A Randomized ablation-based rhythm-control versus rate-control trial in patients with heart failure and atrial fibrillation: results from the RAFT-AF trial. Circulation. (2022) 145(23):1693–1704. 10.1161/CIRCULATIONAHA.121.057095 35313733

[14] Weinmann K Aktolga D Pott A Bothner C Rattka M Stephan T Impact of re-definition of paroxysmal and persistent atrial fibrillation in the 2012 and 2016 European society of cardiology atrial fibrillation guidelines on outcomes after pulmonary vein isolation. J Interv Card Electrophysiol. (2021) 60(1):115–23. 10.1007/s10840-020-00710-4 32124151 PMC7846547

[15] Schönbauer R Duca F Kammerlander AA Aschauer S Binder C Zotter-Tufaro C Persistent atrial fibrillation in heart failure with preserved ejection fraction: prognostic relevance and association with clinical, imaging and invasive haemodynamic parameters. Eur J Clin Invest. (2020) 50(2):e13184. 10.1111/eci.13184 31732964 PMC7027581

[16] Chen S Pürerfellner H Meyer C Acou WJ Schratter A Ling Z Rhythm control for patients with atrial fibrillation complicated with heart failure in the contemporary era of catheter ablation: a stratified pooled analysis of randomized data. Eur Heart J. (2020) 41(30):2863–73. 10.1093/eurheartj/ehz443 31298266

[17] Pan KL Wu YL Lee M Ovbiagele B. Catheter ablation compared with medical therapy for atrial fibrillation with heart failure: a systematic review and meta-analysis of randomized controlled trials. Int J Med Sci. (2021) 18(6):1325–31. 10.7150/ijms.52257 33628087 PMC7893556

[18] Mujović N Marinković M Lenarczyk R Tilz R Potpara TS. Catheter ablation of atrial fibrillation: an overview for clinicians. Adv Ther. (2017) 34(8):1897–917. 10.1007/s12325-017-0590-z 28733782 PMC5565661

