# Supplementary figures and images for: Outcomes of catheter ablation vs. medical treatment for atrial fibrillation and heart failure: a meta-analysis
Source: Front Cardiovasc Med. 2023 May 10;10:1165011. doi: 10.3389/fcvm.2023.1165011 (PMC10206232; doi:10.3389/fcvm.2023.1165011)

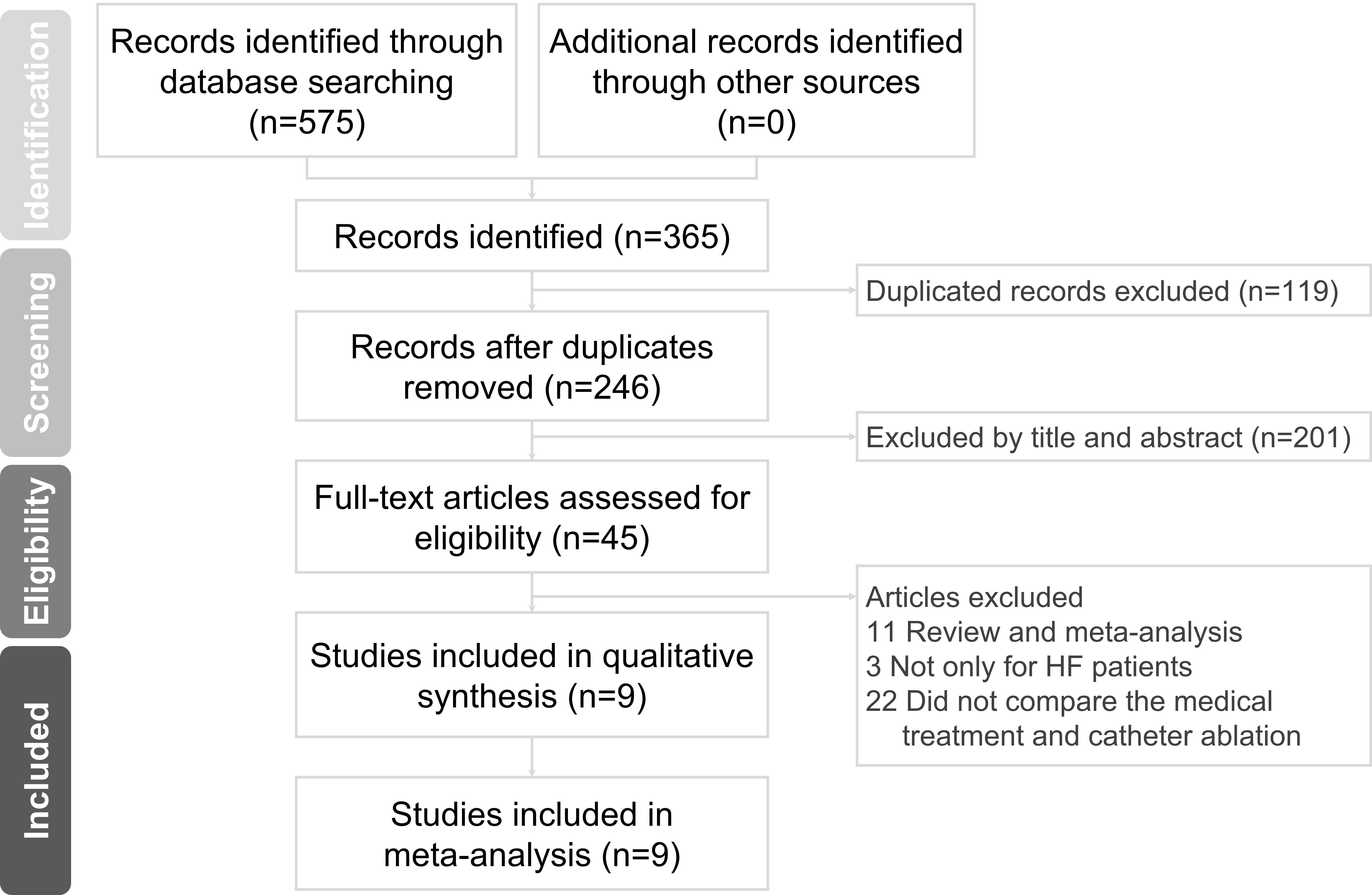

Supplement: Supplementary file 1 [file Image1.jpg]

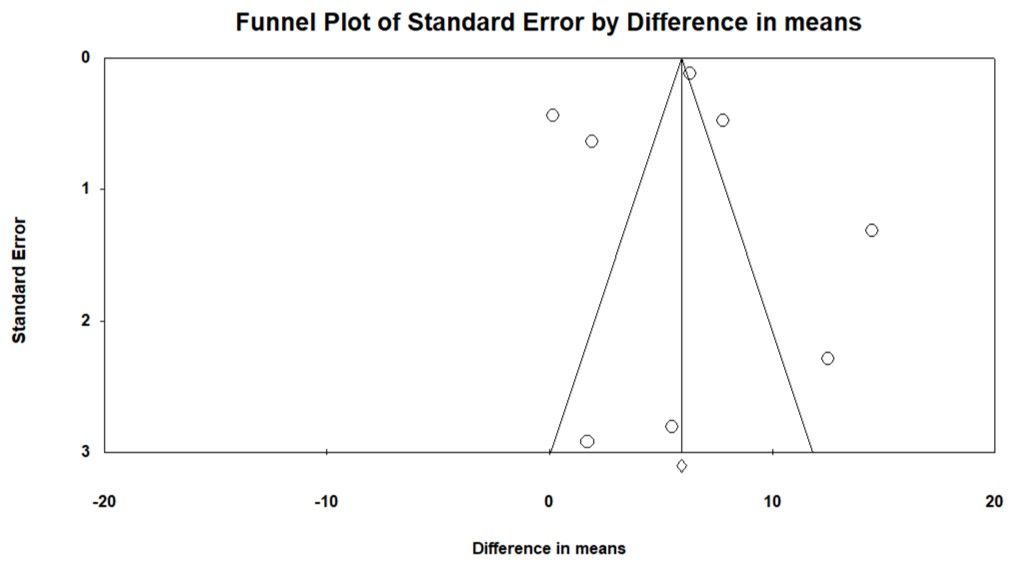

Supplement: Supplementary file 2 [file Image2.jpg]

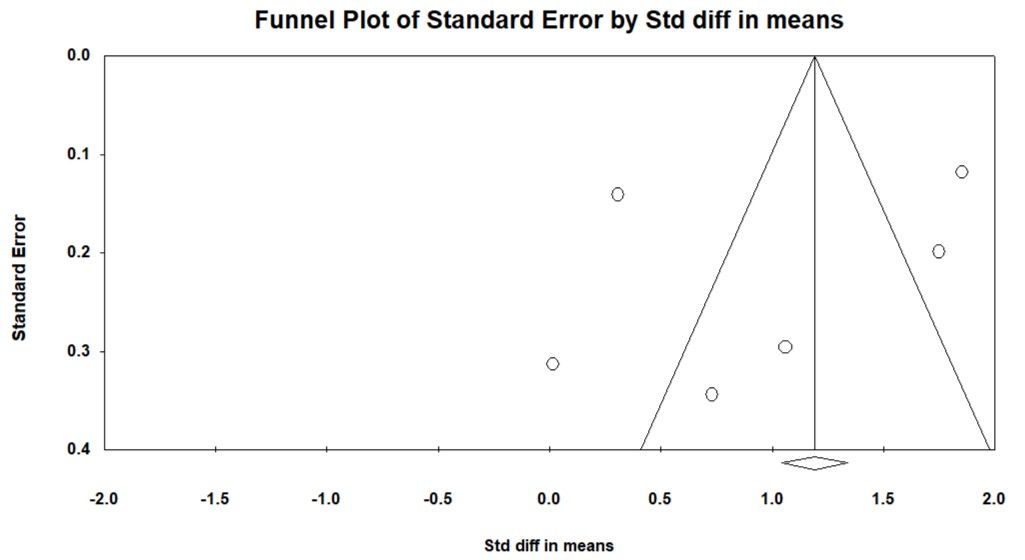

Supplement: Supplementary file 3 [file Image3.jpg]

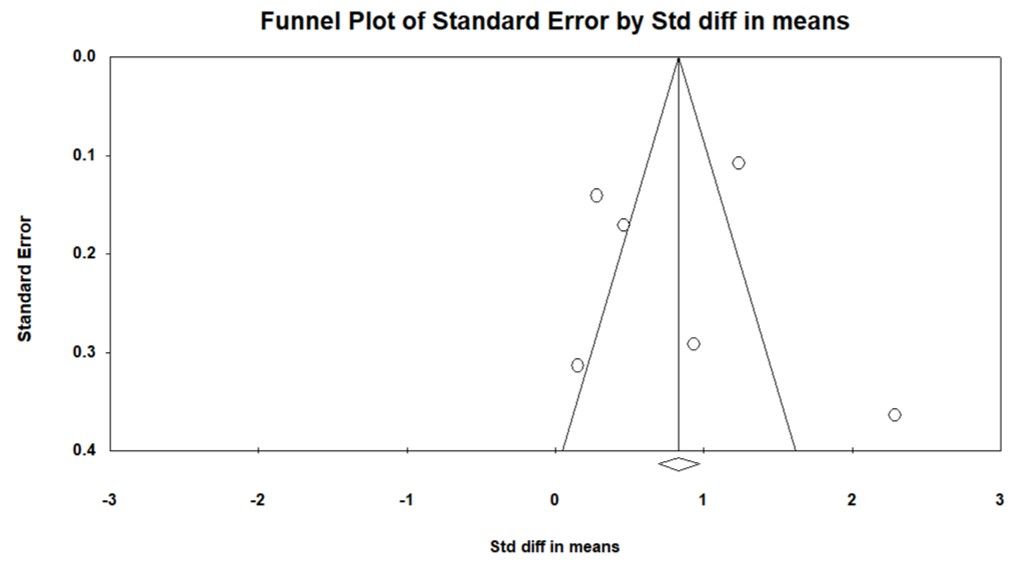

Supplement: Supplementary file 4 [file Image4.jpg]

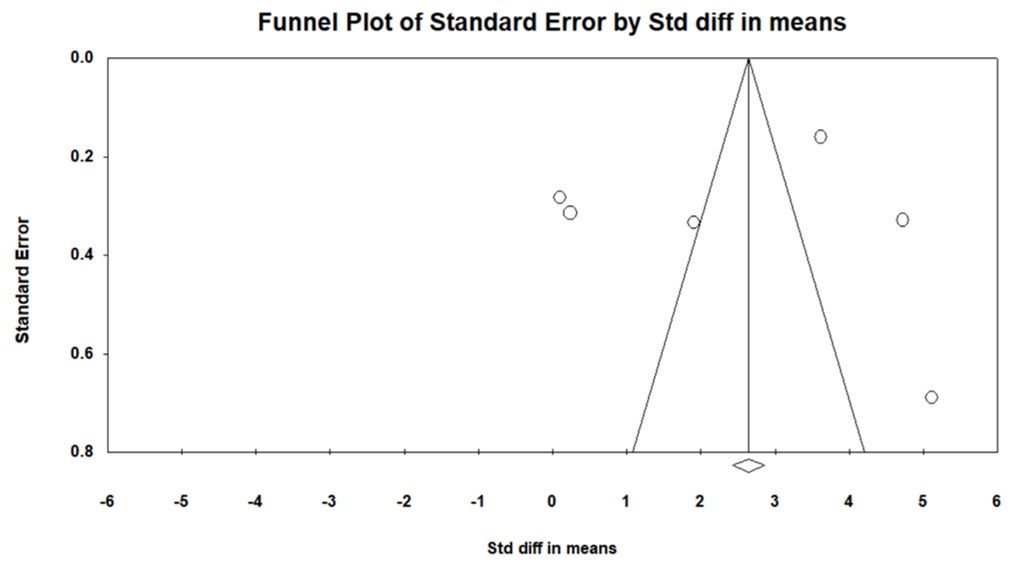

Supplement: Supplementary file 5 [file Image5.jpg]

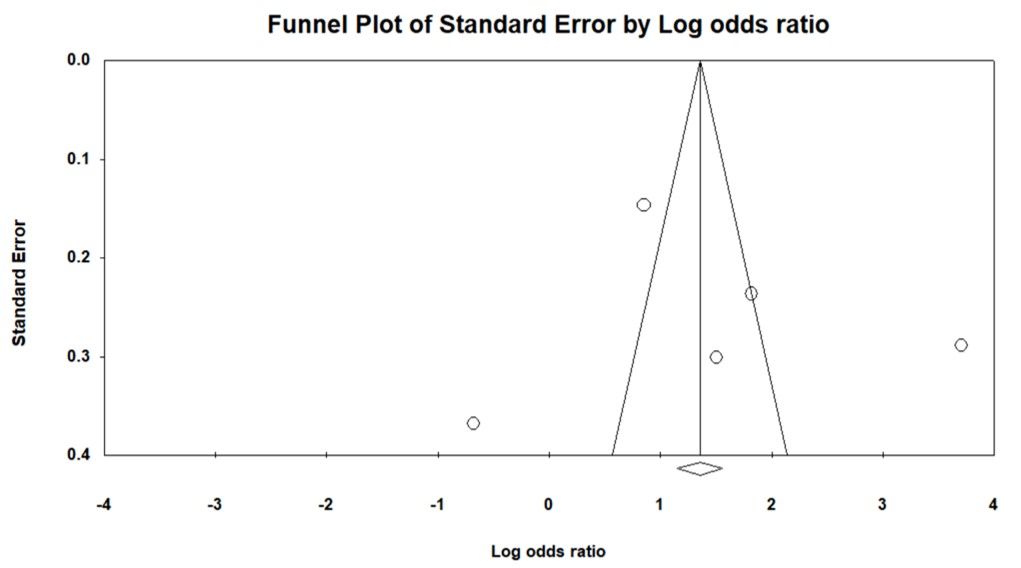

Supplement: Supplementary file 6 [file Image6.jpg]

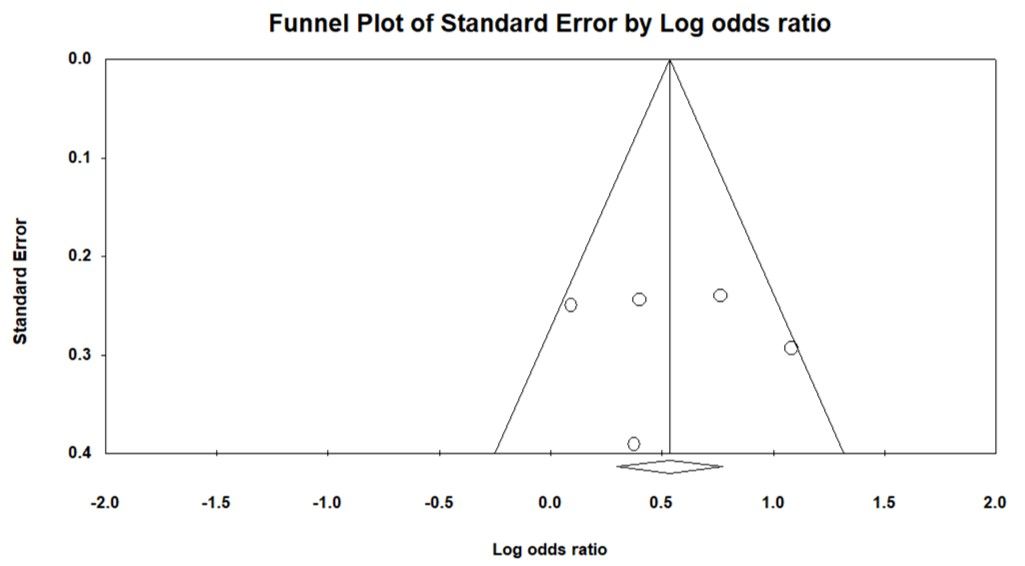

Supplement: Supplementary file 7 [file Image7.jpg]

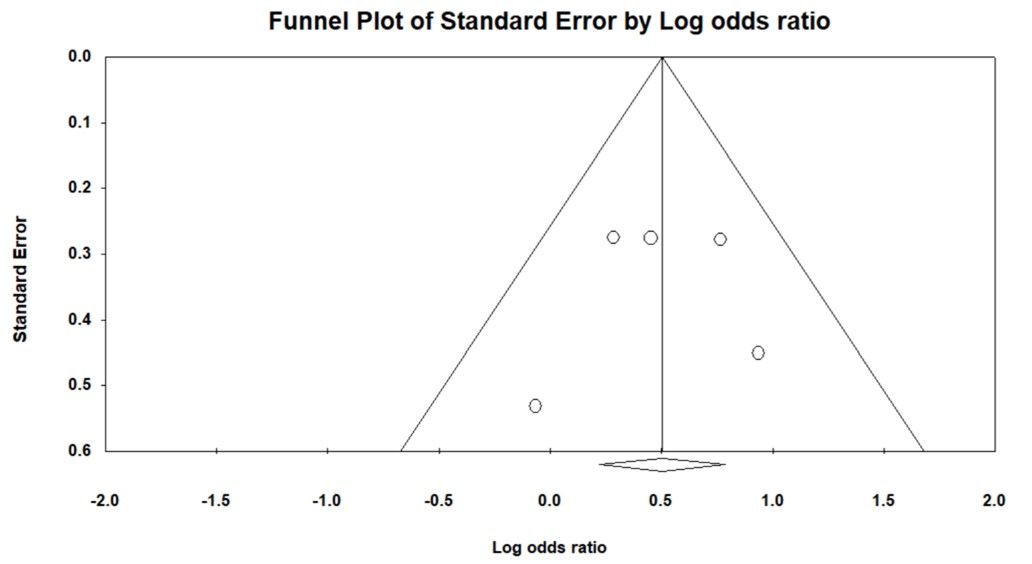

Supplement: Supplementary file 8 [file Image8.jpg]
